# Longitudinal Changes in Self-Reported Walking Ability in Multiple Sclerosis

**DOI:** 10.1371/journal.pone.0125002

**Published:** 2015-05-01

**Authors:** Robert W. Motl, Norman Putzki, Lara A. Pilutti, Diego Cadavid

**Affiliations:** 1 Department of Kinesiology and Community Health, University of Illinois at Urbana-Champaign, Urbana, Illinois, United States of America; 2 Clinical Development Group, Biogen, Cambridge, Massachusetts, United States of America; Charité University Medicine Berlin, GERMANY

## Abstract

**Background:**

Patient-reported outcomes are increasingly used to understand the clinical meaningfulness of multiple sclerosis disability and its treatments. For example, the 12-item Multiple Sclerosis Walking Scale (MSWS-12) measures the patient-reported impact of the disease on walking ability.

**Objective:**

We studied longitudinal changes in walking ability using the MSWS-12 in a cohort of 108 patients with relapsing-remitting multiple sclerosis and moderate-to-severe disability from a single US center cohort study investigating multiple sclerosis symptoms and physical activity.

**Methods:**

The MSWS-12 was completed every 6 months over 2 years together with self-reported measures of disease impact on daily life (Multiple Sclerosis Impact Scale) and walking disability (Patient Determined Disease Steps scale).

**Results:**

The results revealed a high frequency of self-reported changes in walking ability at the individual level, affecting approximately 80% of patients for all four time periods. MSWS-12 scores remained stable at the group level for all four time periods. The magnitude of observed changes at the individual level was higher than the proposed minimal clinically important differences of 4 or 6 points and correlated better with Multiple Sclerosis Impact Scale physical scores than psychological scores, but little with self-reported Patient Determined Disease Steps Scale scores.

**Conclusions:**

This novel finding of frequent fluctuations in self-reported walking ability is new and requires further investigation.

## Introduction

One important life-altering consequence of multiple sclerosis (MS) is walking impairment [1,2]. Walking impairment is a major contributor to decreased quality of life, diminished activities of daily living and loss of employment [2,3]. Walking impairment has traditionally been measured in clinical research using the 500-meter walk of the Expanded Disability Status Scale (EDSS) [4]. The 500-meter walk of the EDSS measures onset or change in walking disability based on changes in distance walked using pre-specific thresholds (e.g. 500 m, 300 m, 200 m, 100 m, 20 m, 5 m) with or without the need for unilateral or bilateral walking aid [4]. Although a change in 500-meter walk threshold (e.g. from 500 m to 300 m) is widely accepted as clinically meaningful, this does not mean that a worsening of lesser magnitude is not [5–8]. The minimal clinically important difference (MCID) in walking ability in MS remains an active area of investigation.

Another major approach for objectively measuring walking ability in MS is the Timed 25-Foot Walk (T25FW), one of three components of the MS Functional Composite scale [9,10]. Unlike the 500-meter walk of the EDSS, the T25FW is linear, reliable, and practical to administer. Several studies indicate that a change in T25FW of at least 20% constitutes a “real change” (e.g. a change beyond the noise) based on test-retest variability [11–13]. Although there is no universal consensus on what constitutes a clinically meaningful change in T25FW, several post hoc analyses of MS studies point at this threshold as a clinically meaningful one [12,14,15]. We recently demonstrated in a cross-sectional MS study that small differences in T25FW are associated with clinically meaningful differences in activities of daily living [16].

Another approach to investigate changes in walking ability in MS is self-reporting using patient-reported outcomes (PROs). The best-known PRO for MS walking ability is the 12-item Multiple Sclerosis Walking Scale (MSWS-12) [17–19]. The MSWS-12 was recently used to demonstrate the treatment effects of prolonged-release fampridine (Fampyra; Biogen, Cambridge, MA, USA) in walking impairment [20] in a patient population with severe walking disability (median EDSS score of 6). The cross-sectional correlations between the T25FW (walking speed) and MSWS-12 values are low (−0.20 to−0.30), although relatively stronger between change values (−0.33 to−0.41) [15], suggesting that they measure different aspects of walking. There has been little research on the “natural history” of MSWS-12 changes at the individual level in the real world. Here we studied the changes in walking ability using MSWS-12 scores in patients with relapsing-remitting MS (RRMS) with moderate to severe disability who were taking part in a single US center longitudinal cohort study investigating symptoms and physical activity in persons with MS [21]. The results revealed marked variations at the individual level in the majority of patients and these changes were not apparent when examined at the group level.

## Materials and Methods

### Study population

The analysis included a subset of patients from a longitudinal investigation of symptoms and physical activity in persons with RRMS from a single research site in the United States. The original sample included 269 persons with RRMS, of whom 108 persons with moderate to severe disability were selected using the Patient Determined Disease Steps (PDDS) scale self-report scores between 3 (gait disability) and 6 (need for bilateral support). There were 51, 41, 13, and 3 RRMS patients with PDDS scores of 3, 4 (early cane), 5 (late cane), and 6, respectively. Of those who provided baseline data, 102 (94%), 102 (94%), 98 (91%), and 93 (86%) provided data at follow-up visits 6, 12, 18, and 24 months, respectively; the few dropouts were caused by change in residential addresses. The demographic and clinical characteristics for this sample are provided in Table 1.

**Table 1:** Demographic and clinical characteristics of the sample of patients with RRMS included in the analysis (*N* = 108).

Characteristic	Mean	Median	SD	Range
Age (years)	47.1	47.0	9.2	20–64
Female, *n* (%)	95 (88)			
Disease duration (years)	10.0	9.0	7.2	1–29
PDDS score	3.7	4.0	0.8	3–6
Treated with DMT, *n* (%)	91 (84)			

DMT: disease-modifying therapy; PDDS: Patient Determined Disease Steps scale; RRMS: relapsing-remitting multiple sclerosis; SD: standard deviation

### MSWS-12

The MSWS-12 is a 12-item patient-rated measure of the impact of MS on walking [17]. The items are rated on a 5-point scale from 1 (not at all) to 5 (extremely) and represent walking limitations during the past 2 weeks. The MSWS-12 is scored by summing the 12 item scores, subtracting 12, dividing the difference by 48, and then multiplying by 100. This scales the MSWS-12 score between 0 and 100 (lower scores indicate less walking disability). The MSWS-12 has good evidence for its internal consistency, test-retest reliability, longitudinal invariance, and validity of scores as a measure of walking mobility in MS [17]. The MCID for the MSWS-12 is estimated to be approximately 4 to 6 points [15].

### Multiple Sclerosis Impact Scale (MSIS-29)

The MSIS-29 is a 29-item patient-reported measure of the impact of MS on physical and psychological health status during the past 2 weeks [22,23]. The MSIS-29 contains 20 items that capture the physical impact of MS (items 1–20), and 9 items that capture the psychological impact of MS (items 21–29). The items are rated on a 5-point scale from 1 (not at all) to 5 (extremely). The MSIS-29 physical domain is scored by summing the scores for items 1 to 20, subtracting 20, dividing the difference by 80, and then multiplying the result by 100. The MSIS-29 psychological subscale (PSYCH) is scored by summing the scores for items 21 to 29, subtracting nine, dividing the difference by 36, and then multiplying by 100. This scales the MSIS-29 physical subscale (PHYS) and PSYCH scores between 0 and 100. The MSIS-29 has good evidence for its internal consistency, test-retest reliability, and validity of scores as a measure of physical and psychological health status in MS. The MCID for the MSIS-29 PHYS is estimated to be 8 points [24]; the MCID for the psychological domain has not been established in the literature.

### PDDS

The PDDS scale contains a single item for measuring self-reported disability status using an ordinal scale ranging from 0 (normal) through 8 (bedridden) [25]. This scale was developed as an inexpensive surrogate for the EDSS and scores from the PDDS have been reported to be linearly and strongly related with physician-administered EDSS scores derived from a neurological examination (rho = 0.93) [25]. We recently confirmed that patient-reported PDDS scores are strongly correlated with physician-completed EDSS scores (rho = 0.783) [26].

### Study procedures

The study was approved through expedited review by the University of Illinois at Urbana-Champaign institutional review board and all participants provided written informed consent before participation in data collection. After initial telephone contact, screening for inclusion, and return of required materials (i.e. signed informed consent and confirmation of MS documents), participants were sent the study materials (i.e. instructions and questionnaires) through the United States Postal Service. Participants were asked to complete the questionnaires and then return the study materials through the United States Postal Service using pre-stamped and pre-addressed envelopes. Participants were contacted by telephone and e-mail as a reminder to return the study materials up to three times. Any missing information was collected by telephone immediately following the return of study materials. This same protocol was repeated for each of the 6-month follow-up periods of data collection during 2 years. All participants received $100 total remuneration; this was prorated $20 per return of study materials.

### Data analysis

All analyses were conducted using PASW Statistics, Version 18, release 18.0.0 (SPSS Inc., Quarry Bay, Hong Kong). We initially estimated descriptive and distributional statistics for MSWS-12 scores across all five time points (0, 6, 12, 18, and 24 months). We next examined mean change in MSWS-12 scores over the 24-month time period using a one-way analysis of variance (ANOVA) with repeated measures over time. This was followed by estimating the internal consistency of MSWS-12 scores across all five time points using Cronbach’s coefficient alpha, and test-retest reliability of MSWS-12 scores over periods of 6 months using intraclass correlation coefficients (single measures) along with 95% confidence intervals. The next analysis examined bivariate correlations between MSWS-12 scores and PDDS and MSIS-29 subscale scores across all five time points using Pearson product-moment correlations and Spearman rho rank-order correlations. We then computed change scores for the MSWS-12, PDDS, and MSIS-29 subscales for each 6-month time period (e.g. time 2—time 1) and examined the bivariate correlations between change scores per time point using Pearson and Spearman correlations. We further examined the bivariate correlations between MSWS-12 change scores per time point using Pearson and Spearman correlations. The next series of analyses involved trichotomizing patients into groups classified as improved, stable, and worsened based on MCID cutoff change values of 4 and 6 for interpreting clinically meaningful MSWS-12 change scores for each time point. After this classification, we estimated the frequency of persons per group classified as improved, stable, and worsened per time point. This was followed by an assessment of agreement between the frequency of persons per group classified as improved, stable, and worsened across the successive 6-month time periods. We next examined changes in MSWS-12 scores over the 2-year period for those who were initially classified as improved, stable, and worsened based on MCID cutoff values of 4 and 6 for interpreting meaningful MSWS-12 change scores.

## Results

### Study population

The majority of patients enrolled into the study were female with RRMS. The mean age was 47 years, the mean disease duration was 10 years, and the median disability as determined by the PDDS was 4 (Table 1). Of the 108 participants, 91 (84%) were currently undergoing treatment with a disease modifying therapy, with 42 using interferon beta-1a, 29 using glatiramer acetate, 11 using interferon beta-1b, and nine using natalizumab. There were two, two, one, and one relapses reported between the four 6-month assessment periods (0–6, 6–12, 12–18, and 18–24 months, respectively); all relapses were reportedly managed with steroids.

### Longitudinal change of MSWS-12 scores at the group level

The mean scores, standard deviations, range of scores, and coefficients of variation along with estimates of skewness and kurtosis for MSWS-12 scores at the group level on each of the five time points examined over 2 years are provided in Table 2. There was no change in mean scores over time at the group level (F [4352] = 0.32, *P* = 0.86 by one-way ANOVA). MSWS-12 group scores had good internal consistency with a mean value for coefficient alpha across the five time points of 0.945. The estimates of test-retest reliability for MSWS-12 group scores across 6-month time periods are reported in Table 3. The test-retest reliability was acceptable, although not consistently strong, with a mean intraclass correlation value across the four 6-month time periods of 0.732.

**Table 2:** Descriptive, distributional, and internal consistency characteristics of MSWS-12 scores for five successive time points each separated by 6 months at the group level in the sample of patients with RRMS.

Time point	*n*	Mean	Median	SD	Range	CV	Skewness	Kurtosis	α
Time 1	108	61.3	60.4	22.8	14.6–100	37.2	−0.1	−0.9	0.933
Time 2	102	60.1	61.5	23.2	0–100	38.6	−0.1	−0.8	0.942
Time 3	102	61.9	64.6	24.1	10.4–100	38.9	−0.2	−1.1	0.947
Time 4	98	62.9	64.6	23.5	8.3–100	37.4	−0.4	−0.7	0.946
Time 5	93	62.7	66.7	26.0	6.3–100	41.5	−0.3	−1.1	0.955

α: coefficient alpha; CV: coefficient of variation; MSWS-12: 12-item Multiple Sclerosis Walking Scale; RRMS: relapsing-remitting multiple sclerosis; SD: standard deviation.

**Table 3:** Estimates of test-retest reliability for MSWS-12 scores across successive 6-month time periods in the sample of patients with RRMS.

Time point	*n*	Intraclass correlation coefficient	95% confidence interval
Time 1—time 2	102	0.726	0.620–0.807
Time 2—time 3	100	0.631	0.497–0.736
Time 3—time 4	97	0.775	0.681–0.844
Time 4—time 5	92	0.797	0.708–0.861

MSWS-12: 12-item Multiple Sclerosis Walking Scale; RRMS: relapsing-remitting multiple sclerosis.

### Correlation of MSWS-12 group scores with other measures of physical and psychological function

The bivariate correlations between MSWS-12 scores and other measures of physical (PDDS, MSIS-29 PHYS) and psychological (MSIS-29 PSYCH) function across each of the five time points are provided in Table 4. MSWS-12 scores correlated more strongly with measures of physical function (PDDS, MSIS-29 PHYS) than with the measure of psychological function (MSIS-29 PSYCH).

**Table 4:** Correlation coefficients for MSWS-12 scores with PDDS and MSIS-29 subscale scores across successive 6-month time periods in the sample of patients with RRMS.

Time point	PDDS	MSIS-29 PHYS	MSIS-29 PSYCH
MSWS-12, time 1	0.574 (0.619)	0.681 (0.670)	0.346 (0.322)
MSWS-12, time 2	0.465 (0.489)	0.613 (0.645)	0.329 (0.292)
MSWS-12, time 3	0.590 (0.636)	0.673 (0.694)	0.235 (0.221)
MSWS-12, time 4	0.645 (0.631)	0.704 (0.706)	0.323 (0.319)
MSWS-12, time 5	0.705 (0.723)	0.688 (0.708)	0.355 (0.344)

MSWS-12: 12-item Multiple Sclerosis Walking Scale; PDDS: Patient Determined Disease Steps scale; PHYS: physical subscale; PSYCH: psychological subscale; RRMS: relapsing-remitting multiple sclerosis.

Correlation coefficients are reported as Pearson (Spearman) correlation coefficients.

The bivariate correlations between 6-month changes in MSWS-12 scores and corresponding changes in PDDS, MSIS-29 PHYS, and MSIS-29 PSYCH scores are provided in Table 5. Importantly, the 6-month change scores approximated a normal distribution based on visual inspection and had minimal evidence of extreme skewness (g_1_ range, -0.34–0.49) and kurtosis (g_2_ range, 1.71–3.31). MSWS-12 change scores were consistently and significantly associated with change scores for the MSIS-29 PHYS. In contrast, there was little if any correlation between change scores for the MSWS-12 and PDDS. There was an initially weak correlation between changes in MSWS-12 scores and MSIS-29 PSYCH scores that became stronger over time. We further examined the correlation of changes over 2 years: the 24-month change in MSWS-12 scores was strongly correlated with 24-month changes in MSIS-29 PHYS scores (r = 0.62) and to a lesser extent with changes in MSIS-29 PYSCH (r = 0.48) and PDDS (r = 0.35) scores.

**Table 5:** Correlation coefficients for change in MSWS-12 scores with change in PDDS and MSIS-29 subscale scores across successive 6-month time periods in the sample of patients with RRMS.

Time point	ΔPDDS	ΔMSIS-29 PHYS	ΔMSIS-29 PSYCH
ΔMSWS-12, time 1—time 2	0.195 (0.193)*	0.459 (0.470)*	0.196 (0.250)*
ΔMSWS-12, time 2—time 3	0.007 (0.048)	0.552 (0.510)*	0.330 (0.376)*
ΔMSWS-12, time 3—time 4	0.144 (0.133)	0.565 (0.508)*	0.454 (0.442)*
ΔMSWS-12, time 4—time 5	0.217 (0.181)*	0.559 (0.541)*	0.521 (0.465)*

MSWS-12: 12-item Multiple Sclerosis Walking Scale; PDDS: Patient Determined Disease Steps scale; PHYS: physical subscale; PSYCH: psychological subscale; RRMS: relapsing-remitting multiple sclerosis.

Correlation coefficients are reported as Pearson (Spearman) correlation coefficients.

*Correlation coefficient statistically significant at *P*<0.05.

### Change in longitudinal MSWS-12 scores at the individual level

Next we analyzed changes in MSWS-12 scores over time at the individual level. To do this, we applied cutoffs that have been proposed as consistent with the MCID [27], either 4 or 6 points. Based on the cutoffs, each patient was classified as improved, stable, or worse for each of the four consecutive 6-month time periods. Using a MCID value of 4 points, approximately 40% of persons reported meaningful improvements for each 6-month time period, whereas approximately 40% reported meaningful worsening (Table 6). The same pattern, but not exact frequency, of cases classified as improved, stable, or worsened over the 6-month time periods was observed using a MCID cutoff of 6 points (Table 6). The results of the ANOVA looking for differences in baseline characteristics indicated that people who worsened in MSWS-12 scores over time had lower baseline MSWS-12 scores, regardless of the 4- or 6-point MCID cutoffs; a similar analysis indicated that people who improved in MSWS-12 scores had shorter disease duration.

**Table 6:** Percentage of patients classified as improved, stable, or worsened in MSWS-12 scores across successive 6-month time periods based on MCID values of 4 and 6.

Time point	*n*	Classification	MCID 4	MCID 6
Time 1—time 2	102	Improved	43.1%	36.3%
		Stable	20.6%	30.4%
		Worsened	36.3%	33.3%
Time 2—time 3	100	Improved	42%	37%
		Stable	18%	28%
		Worsened	40%	35%
Time 3—time 4	97	Improved	41.2%	36.1%
		Stable	18.6%	27.8%
		Worsened	40.2%	36.1%
Time 4—time 5	92	Improved	40.2%	38%
		Stable	15.2%	22.8%
		Worsened	44.6%	39.1%

MCID: minimal clinically importance difference; MSWS-12: 12-item Multiple Sclerosis Walking Scale.

We then analyzed whether there was agreement in classification of individual patients as improved, stable, or worsened in MSWS-12 scores across the successive 6-month time periods. The results indicated marked variability in the classification of individual patients across the successive time periods, regardless of an MCID of 4 (S1 Table) or 6 (S2 Table). Patients often switched classification for each subsequent 6-month time period. For example, 64% of the 44 patients who improved in MSWS-12 scores between times 1 and 2 worsened between times 2 and 3. Similarly, a correlation analysis of patients who improved between times 1 and 2 indicated a significant and strong, but inverse association with worsening between times 2 and 3 (r = –0.693).

**Fig 1:**
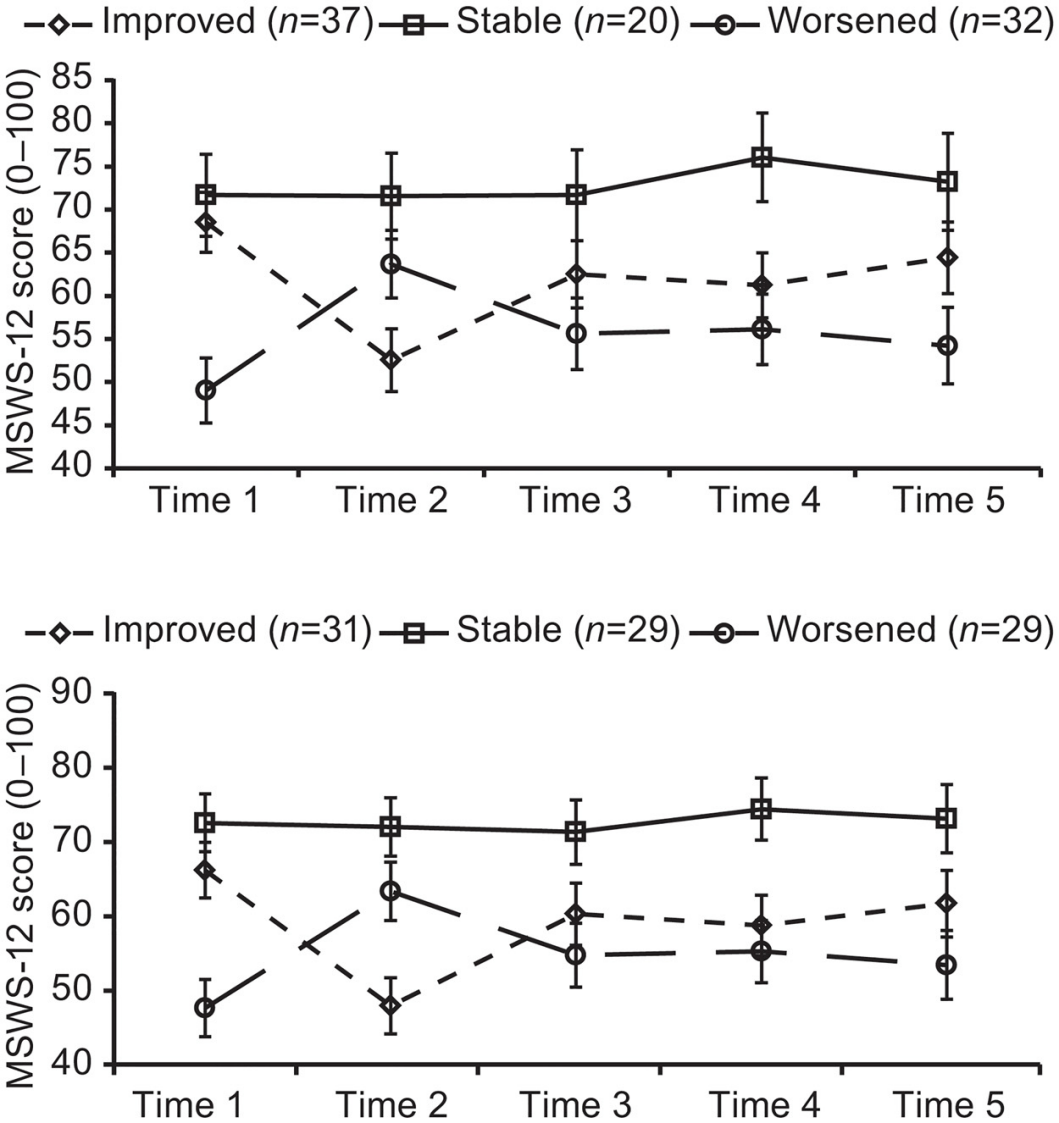
Change in 12-item Multiple Sclerosis Walking Scale (MSWS-12) scores over 2 years. Patients with relapsing-remitting multiple sclerosis were categorized as improved, stable, or worsened across the initial 6-month time period based on minimal clinically important difference values of 4 (top panel) and 6 (bottom panel).

We lastly examined changes in MSWS-12 scores over 2 years for patients classified as improved, stable, or worsened based on change in MCID scores between times 1 and 2. The results demonstrated a group by time interaction on MSWS-12 for MCID values of 4 (F [8344] = 7.42, *P* = 0.001, partial η^2^ = 0.15) and 6 (F [8344] = 7.98, *P* = 0.001, partial η^2^ = 0.16) (Fig 1, mixed-model ANOVA). Importantly, patients initially classified as stable did not have change in MSWS-12 scores over the 2-year period. In contrast, patients initially classified as improved had a large initial improvement in MSWS-12 scores followed by a correction toward baseline values, regardless of the MCID value (Fig 1). Similarly, patients initially classified as worsened had a large initial worsening in MSWS-12 scores followed by an improvement toward baseline, regardless of the MCID. A comparison of changes in MSWS-12 scores across the successive 6-month time periods over 2 years demonstrated significant and strong correlations for consecutive but not for nonconsecutive 6-month time periods (S3 Table).

## Discussion

It is important to have a clear understanding of the patient burden and natural evolution of walking impairment in MS. The MSWS-12 is the leading PRO for measurement of walking ability in MS from the patients’ perspective and complements the established objective assessments (T25FW, EDSS). It has been reported to have excellent reliability and to be more responsive than other patient-based scales such as the Functional Assessment of MS mobility scale and 36-Item Short-Form Health Survey physical functioning scale, and physician-based scales such as the EDSS and Guy's Neurologic Disability Scale lower limb disability item [17]. MSWS-12 standard error of the measure estimates range from 3.9 to 5.1 points (mean 4.5 points). Therefore, using standard error of the measure criteria one can argue that any change of approximately 5 points represent a real change rather than noise [15].

The goal of this study was to investigate the longitudinal changes in self-reported walking ability in a disabled (PDDS range 3–6) yet still ambulatory sample of patients with MS. Patients with progressive MS were not included to avoid the well-known and likely confounding effects of disease progression on MS walking ability, clearly seen over a 2-year period [8]. Very little clinical relapsing activity was observed in this cohort of patients with RRMS, of whom the majority were stable on treatment with approved MS therapies. The expectation was that the patients would be mostly stable. This was clearly not the case, at least based on their self-report of walking ability. The main findings from this analysis are the following: (1) The majority of patients in the study experienced changes in MSWS-12 scores above the 4 or 6 MCID during the successive 6-month time periods examined. (2) There was no meaningful difference in the frequency of change in MSWS-12 scores above the MCID with a cutoff of 6 versus a cutoff of 4. (3) MSWS-12 scores did not change over 2 years at the group level. (4) MSWS-12 scores at the group level correlated much stronger with the PROs of physical function (PDDS, MSIS-29 PHYS) than with the PROs of psychological function (MSIS-29 PYSCH). This extends the current knowledge about the validity of the MSWS-12 as a measurement of physical functioning in a cohort of walking-disabled patients with RRMS. (5) Changes in the MSWS-12 correlated with changes in MSIS-29 PHYS scores, but not with changes in PDSS scores. This suggests that the self-reported PDDS, similar to the EDSS, is insensitive to change [7].

The observation of the high frequency of apparently stable patients with RRMS reporting changes in MSWS-12 scores above the MCID is intriguing. This may very well reflect the true naturally occurring pattern of fluctuation in walking disability in RRMS over time (i.e. patients are reporting a real change in walking that is not captured by objective measures of walking such as the T25FW). In fact, we recently reported that changes in the MSWS-12 of 4 or 6 points did not correspond with changes in performance, gait, or free-living assessments of walking in MS [28]. Nevertheless, this might also reflect shortcomings in the reliability of those MCID values in the context of an observational study [28]. Another possibility is that reported changes in the MSWS-12 are a manifestation of relapsing disease activity not captured by the traditional relapse measures. Studies using frequent and sensitive brain MRI scanning in RRMS show levels of disease activity 30 times larger than clinical relapses [29–31]. In fact, a recent post hoc analysis of longitudinal objective clinical trial data from two progressive MS clinical trials revealed a high frequency of relapsing-remitting activity that was not captured as traditional MS relapses [8]. It is possible that patient reporting via instruments like the MSWS-12 is actually capturing intermittent disease activity. Another possibility is that some observed changes in the MSWS-12 are an artifact from the initial introduction of the instrument to the study patients. This is unlikely because the same frequency of changes continued to be observed during subsequent 6-month time periods of the study when patients were already familiar with the instrument. A fourth possibility is that some of the changes are the result of therapeutic interventions, for example symptomatic treatments for spasticity (e.g. baclofen), demyelination (4-aminopyridine), deconditioning (physical therapy), or depression (e.g. psychotherapy, antidepressants). If true, one would expect the majority of the changes to occur in the direction of improvement, which was not the case; we observed a similar percentage of patients reporting improvement and worsening (Table 6). An additional option is that MSWS-12 scoring is often impacted by fluctuating factors such as fatigue and mood that are prevalent in MS. Given the 6-month intervals between assessments, it may be that seasonal variations in mood also might contribute to fluctuations in MSWS-12 scores. The stronger correlations of the MSWS-12 with PROs measuring physical function rather than psychological function argue against that possibility. In fact, the correlations of changes in the MSWS-12, PDDS and MSIS-29 PSYCH would indicate any such seasonal variation would apply to those PROs as well. Furthermore, seasonal variations need not simply reflect differences in mood because seasonal differences in brain MRI activity have also been reported in MS [32]. One final possibility is that patients with MS are not reliable reporters of walking ability. This may be caused by cognitive impairment, as this has been shown to hamper the ability of patients to accurately report the impact of MS on their life [33]. However, in a recent study we showed that impairment in cognitive processing speed did not interfere with the ability of patients with MS to report on their walking ability [34].

A clear understanding of the reliability and stability of a PRO is needed for the appropriate interpretation of changes that may or may not be observed in randomized clinical trials and in epidemiological and natural history studies. In this sense, our observation that the majority of disabled but still ambulatory patients with RRMS reported frequent changes in MSWS-12 scores above the proposed MCID of 4 or 6 is pertinent. Previous data on the MCID was derived from populations somewhat different from this one [15]. Further research is needed to confirm this observation and to enable an understanding of the cause or causes underlying the reported changes. Equally important will be to make sure that the 22% to 30% of patients who do not report MSWS-12 score changes above the MCID in every 6-month time period (Table 6) are indeed good reporters of the impact of MS in their walking ability. Unlike non-neurological diseases, one needs to be very careful interpreting information from PROs in MS because accurate reporting on the consequences of MS depends on the integrity of neurological functions that are affected by the disease (e.g. sensory perception, processing speed, memory).

We conclude that the natural history of MSWS-12 scores in ambulatory-disabled patients with RRMS in this US cohort is characterized by a high frequency of changes in scores above the proposed MCIDs of 4 and 6 points at the individual level. This is in marked contrast with the group analysis of total MSWS-12 scores over time which gives the false impression of stability. In this sense, the MSWS-12 is reminiscent of the EDSS, in which analysis of score changes at the population level gives the impression that there is no progression, whereas analysis of confirmed changes at the individual level detects clinically meaningful changes in a substantial number of patients [35].

## Supporting Information

S1 Table Agreement in frequency of patient classification as improved, stable, or worsened across successive 6-month time periods based on a MCID value of 4 for the MSWS-12 in the sample of patients with RRMS (*N* = 108). MCID: minimal clinically importance difference; MSWS-12: 12-item Multiple Sclerosis Walking Scale; RRMS: relapsing-remitting multiple sclerosis.
(DOC)

S2 Table Agreement in frequency of patients classified as improved, stable, or worsened across successive 6-month time periods based on a MCID value of 6 for the MSWS-12 in the sample of patients with RRMS (*N* = 108). MCID: minimal clinically important difference; MSWS-12: 12-item Multiple Sclerosis Walking Scale; RRMS: relapsing-remitting multiple sclerosis. ^a^Percent of patients within previous 6-month time period classification.
(DOC)

S3 Table Correlation coefficients for change in MSWS-12 scores across successive 6-month time periods in the sample of patients with RRMS (*N* = 108). MSWS-12: 12-item Multiple Sclerosis Walking Scale; RRMS: relapsing-remitting multiple sclerosis. Correlation coefficients are r (ρ). *Denotes correlation coefficient statistically significant at *P*<0.05.
(DOC)
