# Supplementary material for: Longitudinal Changes in Self-Reported Walking Ability in Multiple Sclerosis
Source: PLoS One. 2015 May 1;10(5):e0125002. doi: 10.1371/journal.pone.0125002 (PMC4416760; doi:10.1371/journal.pone.0125002)
Supplement: S1 Table — MCID: minimal clinically importance difference; MSWS-12: 12-item Multiple Sclerosis Walking Scale; RRMS: relapsing-remitting multiple sclerosis. (DOC) [file pone.0125002.s001.doc]

**S1 Table.** Agreement in frequency of patient classification as improved, stable, or worsened across successive 6-month time periods based on a MCID value of 4 for the MSWS-12 in the sample of patients with RRMS (*N*=108).

|  |  | **Time point: time 2 – time 3** | | |  |
| --- | --- | --- | --- | --- | --- |
| **Time point: time 1 – time** 2 | Classification | Improved | Stable | Worsened |  |
|  | Improved | 11 (25%) | 5 (11%) | 28 (64%) |  |
|  | Stable | 8 (38%) | 7 (33%) | 6 (29%) |  |
|  | Worsened | 23 (66%) | 6 (17%) | 6 (17%) |  |
|  |  | **Time point: time 3 – time 4** | | |  |
| **Time point: time 2 – time 3** | Classification | Improved | Stable | Worsened |  |
|  | Improved | 11 (27%) | 4 (10%) | 26 (63%) |  |
|  | Stable | 8 (50%) | 3 (19%) | 5 (31%) |  |
|  | Worsened | 19 (50%) | 11 (29%) | 8 (21%) |  |
|  |  | **Time point: time 4 – time** 5 | | |  |
| **Time point: time 3 – time 4** | Classification | Improved | Stable | Worsened |  |
|  | Improved | 5 (14%) | 3 (8%) | 29 (78%) |  |
|  | Stable | 9 (50%) | 7 (39%) | 2 (11%) |  |
|  | Worsened | 22 (61%) | 4 (11%) | 10 (28%) |  |

MCID: minimal clinically importance difference; MSWS-12: 12-item Multiple Sclerosis Walking Scale; RRMS: relapsing-remitting multiple sclerosis.
