# Supplementary material for: Longitudinal Changes in Self-Reported Walking Ability in Multiple Sclerosis
Source: PLoS One. 2015 May 1;10(5):e0125002. doi: 10.1371/journal.pone.0125002 (PMC4416760; doi:10.1371/journal.pone.0125002)
Supplement: S2 Table — MCID: minimal clinically important difference; MSWS-12: 12-item Multiple Sclerosis Walking Scale; RRMS: relapsing-remitting multiple sclerosis. aPercent of patients within previous 6-month time period classification. (DOC) [file pone.0125002.s002.doc]

**S2 Table**. Agreement in frequency of patients classified as improved, stable, or worsened across successive 6-month time periods based on a MCID value of 6 for the MSWS-12 in the sample of patients with RRMS (*N*=108).

|  |  | **Time point: time 2 – time 3** | | |  |
| --- | --- | --- | --- | --- | --- |
| **Time point: time 1 – time** 2 | Classification | Improved | Stable | Worsened |  |
|  | Improved | 6 (16%)^a^ | 9 (24%) | 22 (60%) |  |
|  | Stable | 11 (35%) | 12 (39%) | 8 (26%) |  |
|  | Worsened | 20 (62%) | 7 (22%) | 5 (16%) |  |
|  |  | **Time point: time 3 – time 4** | | |  |
| **Time point: time 2 – time 3** | Classification | Improved | Stable | Worsened |  |
|  | Improved | 10 (28%) | 5 (14%) | 21 (58%) |  |
|  | Stable | 9 (35%) | 7 (27%) | 10 (38%) |  |
|  | Worsened | 14 (42%) | 15 (46%) | 4 (12%) |  |
|  |  | **Time point: time 4 – time 5** | | |  |
| **Time point: time 3 – time 4** | Classification | Improved | Stable | Worsened |  |
|  | Improved | 3 (9%) | 6 (18%) | 24 (73%) |  |
|  | Stable | 11 (44%) | 11 (44%) | 3 (12%) |  |
|  | Worsened | 20 (61%) | 4 (12%) | 9 (27%) |  |

MCID: minimal clinically important difference; MSWS-12: 12-item Multiple Sclerosis Walking Scale; RRMS: relapsing-remitting multiple sclerosis.

^a^Percent of patients within previous 6-month time period classification.
