# Supplementary material for: Longitudinal Changes in Self-Reported Walking Ability in Multiple Sclerosis
Source: PLoS One. 2015 May 1;10(5):e0125002. doi: 10.1371/journal.pone.0125002 (PMC4416760; doi:10.1371/journal.pone.0125002)
Supplement: S3 Table — MSWS-12: 12-item Multiple Sclerosis Walking Scale; RRMS: relapsing-remitting multiple sclerosis. Correlation coefficients are r (ρ). *Denotes correlation coefficient statistically significant at P<0.05. (DOC) [file pone.0125002.s003.doc]

**S3 Table.** Correlation coefficients for change in MSWS-12 scores across successive 6-month time periods in the sample of patients with RRMS (*N*=108).

| **Time point** | **1** | **2** | **3** | **4** |
| --- | --- | --- | --- | --- |
| 1. ΔMSWS-12, time 1 – time 2 | _ |  |  |  |
| 2. ΔMSWS-12, time 2 – time 3 | –0.659 (–0.520)* |  |  |  |
| 3. ΔMSWS-12, time 3 – time 4 | 0.132 (0.142) | –0.400 (–0.370)* |  |  |
| 4. ΔMSWS-12, time 4 – time 5 | –0.185 (–0.184) | 0.387 (0.269)* | –0.704 (–0.614)* |  |

MSWS-12: 12-item Multiple Sclerosis Walking Scale; RRMS: relapsing-remitting multiple sclerosis.

Correlation coefficients are r (ρ).

*Correlation coefficient statistically significant at *P*<0.05.
